# Nutritional status, body composition and chemotherapy dosing in children and young people with cancer: a systematic review by the SIOP nutrition network

**DOI:** 10.1038/s41416-025-03023-3

**Published:** 2025-06-26

**Authors:** Amy L. Lovell, Nthongase Makamo, Gareth J. Veal, Melanie B. Bernhardt, Ronald Barr, Rajul M. Gala, Erin Gordon, Elena J. Ladas, Maya Prasad, Paul C. Rogers, Judy Schoeman, Jeremy S. Slone, Karina Viani, Wim J. E. Tissing, Minke H. W. Huibers

**Affiliations:** 1 Department of Nutrition and Dietetics, The University of Auckland, Faculty of Medical and Health Sciences, Auckland, New Zealand; 2 Starship Blood and Cancer Centre, Starship Child Health, Te Whatu Ora, Te Toka Tumai, Auckland, New Zealand; 3 Baylor College of Medicine Children’s Foundation- Malawi/Texas Children’s Hospital Global HOPE-Malawi, Lilongwe, Malawi; 4 Newcastle University Centre for Cancer, Newcastle upon Tyne, United Kingdom; 5 Department of Pharmacy and Pharmaceutical Sciences, St Jude Children’s Research Hospital, Memphis, TN USA; 6 Department of Pediatrics, McMaster University, Health Sciences Centre, Hamilton, ON Canada; 7 Division of Pediatric Oncology, Tata Memorial Hospital, Homi Bhabha National Institute (HBNI), Mumbai, India; 8 Department of Gastroenterology, Hepatology, and Nutrition, Boston Children’s Hospital, Boston, MA USA; 9 Division of Pediatric Hematology/Oncology/Stem Cell Transplant, Columbia University Irving Medical Center, New York, NY USA; 10 Division of Hematology and Oncology, Department of Pediatrics, Faculty of Medicine, University of British Columbia, Vancouver, BC Canada; 11 Division of Paediatric Oncology and Haematology, Department of Paediatrics and Child Health, Faculty of Medicine and Health Sciences, University of Pretoria, Pretoria, South Africa; 12 Department of Pediatrics and Child Health, Faculty of Medicine and Health Science, Stellenbosch University, Stellenbosch, South Africa; 13 Department of Global Pediatric Medicine, St Jude Children’s Research Hospital, Memphis, MA USA; 14 Instituto de Tratamento do Câncer Infantil (ITACI), Clinics Hospital, School of Medicine, University of São Paulo, São Paulo, Brazil; 15 Princess Maxima Center for Pediatric Oncology, Utrecht, the Netherlands; 16 Global Child Health group, Amsterdam university Centre, Amsterdam, the Netherlands

**Keywords:** Paediatric cancer, Paediatric research

## Abstract

**Abstract:**

Malnutrition (undernutrition or overweight/obesity) might significantly impact the pharmacokinetics and pharmacodynamics of antineoplastic drugs in children and adolescents (<21 years). A comprehensive systematic literature search was performed on MEDLINE (PubMed), EMBASE, Web of Science, Scopus, ProQuest, Cochrane Trials, and Cochrane Reviews. Databases were searched up to 30 September 2024. Of 4186 articles identified, 150 full texts were evaluated and 12 selected for inclusion. Eight additional articles were identified following a panel review and 6 included, resulting in a total of 18 articles for data extraction. Relevant pharmacokinetic parameters were described for mercaptopurine, vincristine, anthracyclines, methotrexate, busulfan, bevacizumab, and crizotinib. Due to the heterogeneity and limited number of studies per antineoplastic drug, formal statistical analysis or meta-analysis was not appropriate. Variations in the definition of nutritional status, dosing strategies, and type of pharmacokinetic analyses were observed; therefore, no dosing recommendations could be made. With the increasing childhood cancer burden in LMIC, high prevalence of undernutrition, and the global burden of childhood obesity, there is an urgent need for more research in this area. Prospective studies should incorporate uniform definitions and standardised pharmacological approaches to optimise treatment options for children with cancer globally.

**Systematic literature review registration:**

PROSPERO: (reference: CRD42023435261)

## Background

Survival rates for childhood cancer now exceed 80% in high-income countries (HIC), largely due to advances in treatment protocols including intensive multi-agent chemotherapy [1]. These intensified protocols increase toxicity and are associated with increased long-term risk of chronic disease for survivors as well as decreased quality of life [2, 3]. The narrow therapeutic window of many anticancer agents makes it imperative to identify the balance between efficacy and toxicity. While clinical data are commonly available in adult patient populations, evidence-based dosing guidelines for anticancer drugs in paediatrics are scarce. In addition, nearly 90% of the global childhood cancer diagnoses are in low-to-middle-income countries (LMIC), and dosing guidelines are generally based on results from clinical trials conducted in HIC with less prevalence of (severe) undernutrition and before the global pandemic of childhood obesity [3–6].

Nutritional status, as defined by the National Cancer Institute, encompasses a person’s weight, height, body composition, biochemistry, clinical considerations, and dietary intake [7]. Associations between nutritional status, including undernutrition [8, 9], sarcopenia [8, 10], and drug deposition or treatment outcome have been reported in adult cancer populations [11]. Paediatric studies have indicated that undernutrition in acute lymphoblastic leukaemia (ALL) patients can lead to high rates of febrile neutropenia and extended hospital admissions, while overweight/obese children with ALL have poorer survival rates [12–14]. Therefore, malnutrition of both obesity and underweight is identified as an independent but potentially modifiable prognostic risk factor [15]. Reports from Central America and Nicaragua documented a significant decrease in event-free survival of undernourished children versus well-nourished children with cancer [16–18]. Therefore, it should be of interest to treating physicians whether dosing strategies should be adjusted for altered drug disposition related to nutritional status to avoid suboptimal outcomes.

The impact of dosing strategies on the body can be explored using pharmacokinetics and pharmacodynamics. Pharmacokinetics reflects the absorption, distribution, bioavailability and excretion of a drug within the body [19]. Pharmacodynamics describes the biochemical and physiological effect of the drug on the body [19]. Obesity affects pharmacokinetic parameters notably through changes in plasma proteins, lipid content, drug-metabolizing enzymes, blood flow, and drug transporters [20]. Most notable is the reduced activity of cytochrome P450 enzymes, which play a crucial role in drug metabolism, and which are altered by body composition, dietary intake, and nutritional status [20]. Variations in body composition (i.e. the distribution of fat mass and lean body mass) can impact anticancer drug distribution, metabolism, and clearance, potentially influencing treatment efficacy and toxicity [21]. However, traditional paediatric chemotherapy dosing methods rely on body surface area (BSA) or weight-based dosing that poorly correlates with body composition [22, 23]. Our understanding of pharmacokinetics and pharmacodynamics regarding objective nutritional status remains limited, despite high rates of undernutrition (25–75%) in LMIC and the rising global burden of childhood obesity [4, 24, 25].

Understanding the impact of nutritional status on the pharmacokinetics and pharmacodynamics of antineoplastic drugs is crucial for optimizing paediatric cancer care. The purpose of this review was to evaluate existing evidence for the impact of nutritional status on antineoplastic pharmacokinetics and pharmacodynamics in children and adolescents with cancer, and whether dosing relative to nutrition status impacts treatment-related outcomes including overall survival, event-free survival, treatment related toxicity and mortality.

## Methods

This systematic literature review was initiated by the International Society of Paediatric Oncology (SIOP) Nutrition Network, led by the Steering Group (ALL, RG, MP, JS, MP, JS, KV, MH) with invited experts in the field (NM, GV, MB, RB, EG, EL, PR, WT). The review received funding from SIOP with a goal of interrogating the evidence on the impact of nutritional status on antineoplastic pharmacokinetic and pharmacodynamic parameters and if possible, develop practical evidence-based strategies for treating physicians to optimise paediatric cancer care according to nutritional status. This systematic literature review was guided by the Preferred Reporting Items for Systematic Reviews and Meta-analysis (PRISMA) statement [26] and the protocol was prospectively registered on Prospero (reference: CRD42023435261). The search strategy was developed with the assistance of a library specialist. A comprehensive literature search was performed on MEDLINE (PubMed), EMBASE, Web of Science Core Collection, Scopus, ProQuest Health, Cochrane Trials, and Cochrane Reviews. Medical Subject Headings, major topics, and multi-purpose terms were developed to identify studies in children and young people with cancer who received antineoplastic therapy where pharmacokinetic and pharmacodynamic parameters were assessed according to nutritional status. The reference lists of all relevant articles such as narrative or systematic reviews were searched and original articles where eligible included. The final search was completed on 30 September 2024. Supplementary Table 1 shows the full search strategy presented as PICO criteria.

### Study selection

The inclusion criteria were developed a priori. Studies were included if participants were <21 years of age, with a childhood cancer diagnosis [27, 28], treated with chemotherapy (antineoplastic agents). Nutritional status was defined as being underweight or overweight/obese or using measures such as fat mass/fat-free mass. All study designs were included. No restrictions on the date of publication or language were made.

All retrieved articles were uploaded to a Rayyan database [29] and any duplicates were removed. Title and abstract screening were performed in a blinded, and standardized manner by two independent reviewers. Any publication that was considered relevant on consensus was retrieved for full-text review. Full-text reviews were independently assessed for eligibility by the two reviewers and inclusion was determined via agreement, with verification by a third and fourth reviewer.

### Data extraction and synthesis

Following data extraction and verification, discrepancies were resolved by consensus. The primary outcomes were the impact of covariates of nutritional status (undernutrition or overweight/obesity) on antineoplastic pharmacokinetics or pharmacodynamics. Secondary outcomes included reporting of overall survival, event-free survival, toxicity and adverse events. Data were extracted into a database with variables determined a priori.

### Quality assessment

Study quality was assessed using the risk of bias assessment of observational studies criteria from the International Guideline Harmonisation Group [30, 31] and the Grades of Recommendation, Assessment, Development and Evaluation (GRADE) criteria [32, 33] to assess the quality of evidence for each antineoplastic drug. Quality assessment was conducted by two independent reviewers.

## Results

**Fig. 1:**
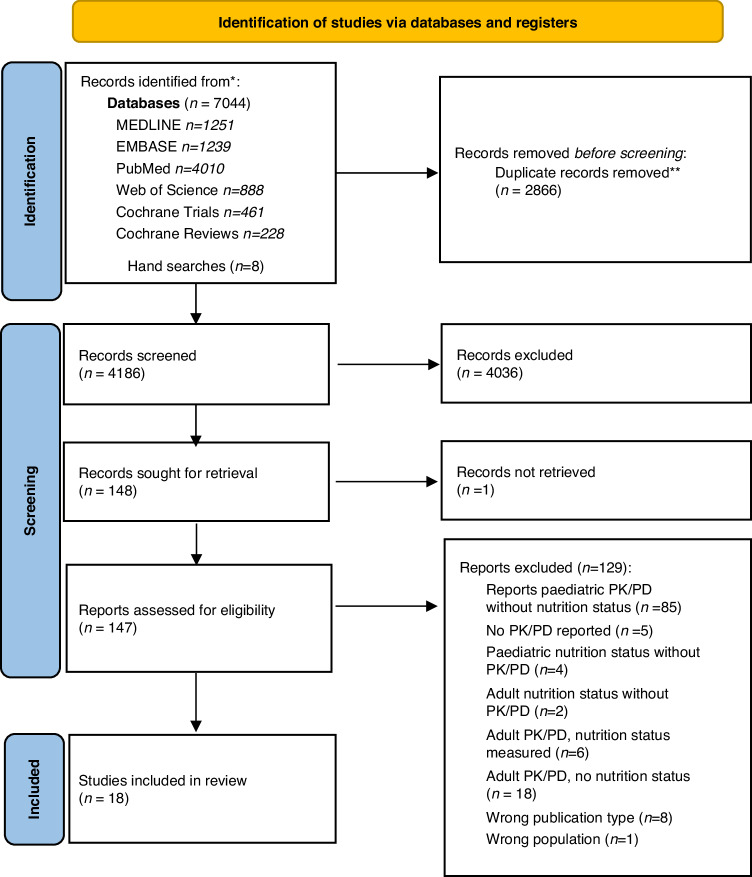
PRISMA flowchart for the selection of the original studies on the association between nutritional status and antineoplastic pharmacokinetics and pharmacodynamics in children and adolescents (<21 years) with cancer. * Number of records identified from each database searched. **Duplicates excluded using Rayyan software [29].

A total of 4186 potential articles were screened, with 150 individual studies meeting the inclusion criteria for full-text review, and 12 articles being eligible for inclusion. Eight further articles were identified following discussion on the third and fourth reviewer, of which six were eligible for inclusion, resulting in 18 articles for data extraction (Fig. 1) [26].

### Study characteristics

Fourteen studies were prospective in their study design [34–47], three were retrospective [48–50], and one study was a case report on a single individual [51]. A total of 1194 children were included across all studies (data population ranged from 1 [51] to 621 [48]). Anthracyclines were the most common antineoplastic investigated, with six studies evaluating doxorubicin [34, 41–43, 46, 51] and one study evaluating daunorubicin [37]. Five studies investigated methotrexate [36, 45, 47, 48, 50], two studies each for busulfan [39, 49], topoisomerase II inhibitors [48, 51], and mercaptopurine [44, 48]. Additional drugs investigated included vincristine [40], crizotinib and dasatinib [35], bevacizumab [38], and cytarabine [48] (Table 1).

**Table 1:** Study and population characteristics for the included studies that evaluated the effect of nutritional status on chemotherapy pharmacokinetics in children and young people <21 years of age.

Author Year of publication	Study design	Sample size	Age, years median (range)	Male:Female *n* (%)	Ethnicity or Race *n* (%)	Cancer type	Chemotherapy	Chemotherapy dose or treatment protocol	Nutrition status *n* (%)	Risk of bias [33]
Egnell et al. [47]	Prospective	182	5 y.^a^	NR	NR	ALL	HD-Methotrexate	5 g/m^2^	Under: 9 (5) Normal: 146 (80) Over: 20 (11) Obese: 7 (4)	SB: high AB: low DB: high CF: low
De Olivieira Henz et al. [50]	Retrospective	45	6.42 (0.33–17.8) y.	27 (60): 18 (40)	Brazilian population	ALL	Methotrexate	0.25-5 g/m^2^	Under: 7 (16) Normal: 26 (57) Over: 7 (16) Obese: 4 (10)	SB: high AB: low DB: high CF: low
Gándara-Mireles et al. [34]	Prospective	70	10.67 (2.8) y.	37 (53): 33 (47)	NR	ALL	Doxorubicin	30 mg/m^2^	BMI as continuous variable	SB: high AB: unclear DB: high CF: low
Gibson et al. [35]	Phase I trial	36	10.6 y. (2.9–21.3)	11 (31): 25 (69)	White: 25 (69)Black: 6 (17) Unknown: 5 (14)	HGG DIPG	Crizotinib Dasatinib	Initial: 130 mg/m^2^ Crizotinib, 50 mg/m^2^ Dasatinib Modified: 165 mg/m^2^ Crizotinib, 50 mg/m^2^ Dasatinib	Under: 0 (0) Normal: 17 (47) Over: 9 (25) Obese: 10 (28)	SB: high AB: low DB: high CF: low
Orgel et al. [36]	Prospective (2° analysis)	36	14.9 y. (10.2–19.2)	22 (61) :14 (39)	NR	HR B-ALL T-ALL	Methotrexate	0.5 mg/m^2^ 4.5 gm/m^2^	Obese BMI%: 6 (17) Obese BFP: 7(19)	SB: high AB: high DB: high CF: low
Thompson et al. [37]	Prospective	98	12 y. (0.5–20.4)	67 (68): 31 (32)	White: 70 (71)Black: 12 (12)Other: 16 (16)	ALL AML Lymphoma Other	Daunorubicin	COG (not specified)	Under: 5 (5) Normal: 65 (66) Over: 12 (12) Obese: 16 (16)	SB: low AB: low DB: high CF: low
Turner et al. [38]	Prospective	27	12.2 y. (6.8–18.1)	16 (59): 11 (41)	White: 15 (56)Black: 9 (33) Hispanic: 2 (7)Asian: 1 (4)	Osteosarcoma	Bevacizumab	15 mg/kg	Under: 3 (1) Normal: Over: 11 (41) Obese: NR	SB: high AB: low DB: high CF: low
Browning et al. [39]	Prospective	37^b^	7.1 (6.1) y^c^	41 (60): 27 (40)	NR	Leukaemia Lymphoma Solid	Busulfan	0.8 mg/kg (AIBW)	Under: 11 (30) Normal: Over: 18 (49) Obese: x	SB: high AB: low DB: high CF: low
Israels et al. [40]	Prospective	19	15 y. (3.3–21.5)	16 (84)	Malawi 11 (58) UK:8 (42)	Wilm’s	Vincristine	1.5 mg/m^2^	Presented as continuous z-score	SB: high AB: low DB: high CF: low
Thompson et al. [46]	Prospective	22	15 y. (3.3–21.5)	16 (73): 6 (27)	Hispanic: 10 (45) Caucasian: 10 (45) Asian: 2 (10)	HL ALL BL NHL Osteosarcoma NBL SS Hepatoblastoma	Doxorubicin	NR	Under: 5 (23) Normal: 15 (68) Over: 2 (9)	SB: low AB: low DB: high CF: low
Ritzmo et al. [51]	Case report	1	14 y.	1 male	NR	Hodgkin Lymphoma	Doxorubicin Etoposide	OEPA course in GPOH-HD 2002	Obese: 46.3 kg/m^2^	SB: high AB: low DB: high CF: unclear
Hijiya et al. [48]	Retrospective	621	NR	341 (55): 279 (45)	White: 495 (80) Black: 91 (14) Other 35 (6)	ALL	Methotrexate Etoposide Teniposide Cytarabine Mercaptopurine	St Jude (Total XII, XIIIA, XIIIB, and XIV)	Under: 102 (16) Normal: 400 (64) Over: 64 (11) Obese: 55 (9)	SB: low AB: low DB: high CF: low
Hempel et al. [41]	Prospective	27	4.12 y. (1.56–19.99)	12 (44): 15 (56)	NR	NHL ALL	Doxorubicin	ALL-BFM 95 NHL-BFM 95 30 mg/m^2^	BMI: 16.7 (13.7-22.2)	SB: high AB: low DB: high CF: low
Frost et al. [42]	Prospective	107 ^d^	4.8 y. (1.3–17.3)	63 (59): 44 (41)	NR	ALL	Doxorubicin	NOPHO ALL-92	BMI: 16.3 (12.6 – 25.6)	SB: low AB: low DB: high CF: low
Dupuis et al. [49]	Retrospective	38^e^	5.7 y. (0.17–17.5)	20 (53): 18 (47)	NR	AML CML Histiocytosis Soft tissue sarcoma HL	Busulfan	40 mg/m^2^	Calculated actual weight vs. ideal weight.	SB: high AB: low DB: high CF: unclear
Eksborg et al. [43]	Prospective	31	5.4 y. (0.73–15.3)	18 (58): 13 (42)	NR	ALL	Doxorubicin Epi-doxorubicin	NOPHO SR/IR protocol NOPHO HR protocol	NR	SB: high AB: low DB: high CF: unclear
Zuccaro et al. [44]	Prospective	18	6.9 y. (3–15)	9 (50) : 9 (50)	NR	SR ALL	Mercaptopurine	75 mg/m^2^	Normal: 9 (50) >75^th^ %tile: 9 (50)	SB: high AB: low DB: high CF: unclear
Kumar et al. [45]	Prospective	6	1–15 y.	4 (67): 2 (33)	Asian	Unspecified malignancies	Methotrexate	50 mg/m^2^	Relative weight <90%	SB: high AB: low DB: high CF: unclear

*AB* Attrition Bias, *AIBW* adjusted ideal body weight, *ALL* acute lymphoblastic leukaemia, *AML* acute myeloid leukaemia, *BFP* body fat percentage, *BMI* body mass index, *CF* Confounding, *DB* Detection Bias, *DIPG* Diffuse intrinsic pontine glioma, *HGG* high-grade glioma, *HL* Hodgkin Lymphoma, *HR* high-risk, *HSCT* Hematopoietic stem cell transplantation, *IR* intermediate risk, *LBM* lean body mass, *NBL* Neuroblastoma, *NHL* Non-Hodgkin lymphoma, *NR* not reported, *SB* Selection Bias, *SR* standard risk, *SS* Synovial sarcoma.

^a^Only reported mean age.

^b^Total pop 68 (only 37 had a malignant diagnosis).

^c^Only reported mean(SD).

^d^*n* = 5 were infants <1 y. Not included in other stats.

^e^Also included Non-malignant (metabolic storage disease, immune deficiency, congenital neutropenia).

### Definition of nutritional status

Different definitions of nutritional status were used across the studies (Supplementary Table 2). Seven studies used body mass index (BMI) as a continuous variable [34, 36, 42–45, 51], and two studies used an arbitrary cut off of </>19.4 kg/m^2^ [34] and </>75th percentile [44] to group participants. In the six studies [35, 37–39, 46, 51] that used the Centre for Disease Control (CDC) growth charts [52, 53] the classification of under-and overnutrition according to BMI-for-age varied, with three studies defining undernutrition as ≤10th percentile [37, 45, 46], two studies as ≤5th percentile [35, 38] and one as <25th percentile [39]. All studies except one [39] defined obesity as ≥95th percentile. One study expressed nutritional status as a standardized continuous z-score [40], one [50] used WHO z-score categories [54], and one used BMI cut-offs according to Cole et al. for underweight and International Obesity Task Force guidelines for overweight and obesity [55, 56]. Body composition (percentage body fat) determined via dual energy x-ray absorptiometry scan was reported in three studies [36, 37, 46]. No studies measured arm anthropometry using mid upper arm circumference (MUAC).

### Chemotherapy dosing & pharmacokinetic analysis

Antineoplastic drugs were largely dosed using BSA among children (*n* = 16, 94%) [34–38, 40–50]. One study used adjusted ideal body weight (*n* = 1, 6%) [39]. Where BSA was calculated, twelve studies used actual body weight [34–38, 40–45, 48–50], one used adjusted body weight [51], one used ideal body weight [38], and in two studies the use of actual body weight was unclear [34, 35]. For overweight/obese children, there was variation in the methods used for calculating drug dose, with different studies using both adjusted [51] and ideal body weight [38] approaches. All antineoplastics were administered via IV infusion, apart from busulfan (orally or via a nasogastric tube) [49], crizotinib (orally), dasatinib (orally) [35], and Mercaptopurine (orally) [44, 48].

No studies reported pharmacodynamic analyses. Pharmacokinetic parameters were assessed by compartmental [34, 35, 37, 41, 46, 50] or non-compartmental analysis [38, 40], as single-dose pharmacokinetic studies [35, 39], limited sampling models [42, 51], and using standard formulae [44, 45, 49]. Three studies [34, 37, 50] evaluated the effect of genetic polymorphisms on pharmacokinetic parameters. Drug clearance was the most reported pharmacokinetic parameter and was detailed in thirteen studies [34, 35, 37–39, 42, 44–48, 50, 51]. Eight studies reported on drug volume of distribution [34, 35, 37, 38, 44–46, 50]. Six studies reported on dose elimination and clearance via area under the plasma concentration-time curve (AUC) method [35, 36, 39, 40, 44, 49], and six studies reported maximum plasma concentrations over time [35, 41–44, 51]. Elimination half-life was reported in three studies [44, 45, 51], and one study reported each on the rate of elimination [36] and absorption rate constant [35].

### Impact of undernutrition on pharmacokinetics

Eleven studies reported on the effect of undernutrition on antineoplastic pharmacokinetics [34, 37–43, 45, 48, 50] (Table 2). Vincristine clearance was compared between patients with Wilms tumour in Malawi (median z-score weight for height = −2.3) versus the United Kingdom (UK) (median z-score weight for Height= 0.4), where vincristine clearance was significantly lower in the Malawian population (*p* = 0.001) [34, 37]. No consistent correlation between BMI or body composition and doxorubicin pharmacokinetics has been reported [34, 41–43, 46, 51]; with two studies reporting a possible association between body fat percentage (≥30%) and clearance [34, 46]. A trend towards higher dose-normalised peak plasma concentrations for doxorubicin was reported in in children with a lower BMI (12.2–16.3 kg/m^2^) compared to those with a higher BMI (16.7–21.5 kg/, BMI measured as continuous measure) [43]. Similarly, undernourished children (BMI < 19.4 kg/m^2^) and age (<7 years) were associated with decreased clearance (r = −0.0873 ml/min, *p* = 0.003 and r = −0.562, *p* = 0.001), respectively and therefore increased exposure in a separate study [34].

**Table 2:** Summary of pharmacokinetic outcomes for each antineoplastic drug and the direction of their association with nutritional status.

Drug	Method	PK parameters	Nutritional Status		
			Undernutrition	Overweight	Obesity
Bevacizumab	Population PK model [37, 38]	V_d_	≥32.1% ↑ [38]		≥53% ↓ [37]
Busulfan	Dosing study [39] Dose-adjustment PK study [49]	Dose	↑↑ [39]	↓ [39]	↓ [39] ↔[49]
		CL		↓ [39]	↓ [39]
		AUC		↑ [39]	↑ [39]
Crizotinib	Steady state PK model [35] Two-compartment model [35]	C_max_		1.6-fold ↑ [35]	1.6-fold ↑ [35]
		CL		↓ [35]	↓ [35]
		AUC		1.6-fold ↑ [35]	1.6-fold ↑ [35]
		V_1_/F		↓ [35]	↓ [35]
Cytarabine	One-compartment model [48]	CL	↔ [48]	↔ [48]	↔ [48]
Daunorubicin	Two-compartment model [37] (One-compartment model: Daunorubicinol) [37]	CL	↔ [37]	↔ [37]	↔ [37]
		V_d_	↔ [37]	↔ [37]	↔ [37]
Doxorubicin	Two-compartment model [34] Three-compartment model [46] Limited sampling model [42, 43, 51]	C_max_	↔ [42] ↔ [41] ↑ [43]^b^	↔ [42] ↔ [41]	↔ [42] ↔ [41]
		CL	↓ [34]		↓ [46] ^c^ ↔ [51]
		V_d_			↓ (Doxorubicinol) [46] ^c^
Epi-doxorubicin (Epirubicin)	Limited sampling model [43]	C_max_	↑ [43]^b^		
Etoposide	Limited sampling model [51] Two-compartment model [48]	CL	↔ [48]	↔ [48]	↔ [51] ↔ [48]
Mercaptopurine	Standard equations [44] Two-compartment model [48]	C_max_		↓ [44]^d^	↓ [44]^d^
		CL	↔ [48]	↑ [44] ^d^ ↔[48]	↔[48]
		AUC	↑ [44] ^d^		
		V_d_		↑ [44] ^d^	
Methotrexate	Non-parametric population PK model [36] Two-compartment PK model [48, 50] Standard formula [45, 47]	CL	↔ [48] ↓ [50]	↔ [48]	↔ [48] ↑ [47]
		AUC	↔ [36]	↔ [36]	↔ [36]
		k			2-fold ↓ [36] ↓ [45]
Teniposide	Two-compartment model [48]	CL	↔ [48]	↔ [48]	↔ [48]
Vincristine	Non-compartmental analysis [40]	CL	↓ [40]		
		AUC	1.98-fold ↑ [40]^a,e^		

↑, increased; ↓, decreased; ↔, no effect.

*ABM* adjusted body measurement, *AUC* area under the curve, *CL* clearance, *C*_*max*_ maximum plasma concentration, *k* rate of elimination, *PK* pharmacokinetic, *TBM* total body mass, *V*_*d*_ volume of distribution, *V1/F* volume of central distribution.

^a^Test dose used for PK comparison.

^b^Dose normalised.

^c^BF ≥ 30%.

^d^<75th percentile.

^e^Body weight adjusted for tumour weight.

For busulfan, children with extreme undernutrition (BMI for age <5th percentile), required the highest dose per m^2^ during haematopoietic stem cell transplant conditioning (for malignant and non-malignant disorders) to achieve the same AUC as normal or obese children (with a high BMI) [39]. Similarly, for bevacizumab, children <3rd percentile- weight for age, the volume of distribution was up to 32% higher compared to children with normal nutritional status [38].

Kumar et al. found a significant negative correlation (−0.70) between relative weight and the elimination half-life of methotrexate in six Indian patients with ALL [45], and de Oliveira Henz et al. reported reduced clearance only in children classified as underweight relative to age from Brazil [50]. No effect of undernutrition on (systemic) drug clearance was reported by Hijiya et al. for cytarabine, etoposide, mercaptopurine, or teniposide in 621 children with ALL treated on the St. Jude Total Therapy studies [48].

### Impact of overweight and obesity on pharmacokinetics

Twelve studies reported on the effect of overweight and obesity on pharmacokinetic parameters [35–37, 39, 41–44, 46, 48, 51] (Table 2). For mercaptopurine, overweight patients (BMI >75th percentile) had a higher drug clearance and volume of distribution, resulting in a lower AUC and maximum plasma concentration [44]. A significant linear correlation was observed for this drug, where those with higher fat mass exhibited the lowest plasma concentration of mercaptopurine [44].

Orgel et al. [36] reported a 2-fold increased risk of delayed methotrexate elimination in obese children (*n* = 6), defined as percentage body fat >45% [36]. However, using a BSA-adjusted population pharmacokinetic model, neither percentage body fat nor BSA was linearly associated with AUC for high-dose methotrexate. Interestingly, when body fat percentage was held constant, children with a higher BSA (i.e. a larger body size) experienced a similar increase in risk of delayed elimination [36]. More recently, an increased risk of prolonged excretion in children who were obese at diagnosis was reported in a Dutch population, with a decrease in BMI standard deviation score before the first course being independently associated with altered (increased) excretion [47]. When only using BMI to classify nutritional status, Hijiya et al. did not find any relationship between obesity and methotrexate clearance [48].

The impact of overweight and obesity on anthracycline pharmacokinetics was reported in six studies [37, 41–43, 46, 51]. No effect of increased body fat (>30%) or obesity (BMI for age percentile >95th) on the pharmacokinetics of daunorubicin were reported by Thompson et al. [37]. For doxorubicin, the same group reported that a body fat percentage greater than 30% resulted in lower clearance and volume of distribution, which became statistically significant for its metabolite, Doxorubicinol [46]. This relationship was also observed for two obese patients (BMI for age percentile >85th)) [46]. Using BMI or lean body mass as a continuous variable, there was no correlation with BSA-normalised peak plasma concentrations of doxorubicin [43]. In a single case study, plasma clearance of doxorubicin in an obese child (BMI 46.3 kg/m^2^) was comparable with non-obese children in the literature [51].

For busulfan, Dupuis et al. reported no effect of obesity on dose requirements (dose based on BSA by actual weight) [49]. However, Browning et al. reported higher drug exposures for Busulfan when doses were calculated using actual weight for obese children (BMI-for-age ≥85th percentile). These children also required a smaller drug dose to achieve the same AUC as children with normal nutritional status [39]. When compared to the recommended dosing equation, the use of ideal body weight to determine dose in obese children ≥85th percentile (BMI-for-age), resulted in an increased risk of over or underdosing children by ≥20% in 53% of children in that category [39]. Similarly, crizotinib clearance and volume of distribution in overweight/obese children compared to children with normal nutritional status were significant lower (44.2 L/h/m^2^ versus 75.5 L/h/m^2^; *p* = 0.0015 and 75.5 vs. 119.3 L/m^2^;*p* < 0.0001) [35]. Comparable outcomes were shown by a non-linear relationship between total body weight and bevacizumab volume of distribution. For obese children (BMI-for-age >97th percentile) the volume of distribution was up to 53% lower when compared to the population median [38]. No effect of overweight or obesity on antineoplastic clearance were reported by Hijiya et al. for cytarabine, etoposide, mercaptopurine or teniposide, all dosed per m^2^, in 621 children with ALL treated on the St. Jude Total Therapy studies [48].

### Outcomes

Four studies reported on outcomes such as toxicity, remission or relapse and survival [34, 36, 44, 48]. Children with wasting (BMI < 19.4 kg/m^2^) experienced a decrease in the intercompartmental clearance of doxorubicin and increased AUC values; the effect of which was a prolonged effect of the drug and associated increased risk of cardiotoxicity with systolic and diastolic dysfunction [34]. In evaluating toxicities associated with high-dose methotrexate, Orgel et al. [36] did not report their findings according to nutritional status; however, no association between AUC and toxicity was found [36]. When using BMI only to classify children into categories of nutritional status, Hijiya et al. did not find any difference between groups for remission, relapse, overall survival, event-free survival, and Grade III and IV toxicities [48]. Two overweight children (BMI-for-age >75th percentile) showed decreased mercaptopurine concentrations and relapsed after maintenance treatment [44].

### Study quality

The GRADE criteria [32, 33] were used to determine the quality of evidence of the 16 studies, and 14 drugs, included in this review. The overall quality of evidence was very low (Supplementary Table 3).

## Discussion

Undernutrition and overweight/obesity in paediatric cancer patients have the potential to alter antineoplastic drug pharmacokinetic parameters including distribution and excretion [23, 25]. This systematic review is the first to examine the association of the spectrum of nutritional states on antineoplastic pharmacokinetics. Determining the implications of findings was challenging due to varied definitions of altered nutritional status and heterogeneity in dosing strategies and pharmacokinetic analyses performed.

### Drug dosing

Key considerations in paediatric cancer treatment include normalizing or scaling of nutrition and pharmacokinetic parameters for patient size, particularly when nutritional status is superimposed on normal age-related variation in child size [57]. While BSA is commonly used for dose-normalisation of antineoplastics in adult and paediatric patients [58], its adjustment for ideal or actual body weight as well as dosing methods (BSA, weight-based, capped, flat) for children, vary and lacks consensus [58]. For example, obese children with body fat over 45% were twice as likely to develop delayed methotrexate elimination at 48 h; however, when holding body fat percentage constant, a higher BSA (i.e. larger children) also resulted in delayed elimination (likely due to differences between BSA dosing and the estimation of model pharmacokinetic parameters in humans) [36]. In this instance, severely altered nutrition status might not be adequately reflected in BSA, necessitating future global pharmacokinetic studies that consider differences in body composition for undernourished and obese children with cancer to perform objective and effective pharmacokinetic evaluations [59, 60]. Whilst not reported in the included studies, the impact of chronic stress, inflammation, and other biological effects of the fat free mass to fat mass ratio on the immune [and tumour] response on chemotherapy pharmacokinetic is an important consideration and understudied area of pediatric cancer research.

Drug lipophilicity is an important variable in the context of nutritional status, especially adiposity, and drug distribution. Lipophilic drugs diffuse into adipose tissues rapidly, resulting in an increased volume of distribution, necessitating higher doses for equivalent serum levels compared to less lipophilic antineoplastics [61]. Lipophilic drugs in this review include bevacizumab [38], busulfan [39, 49], crizotinib [35], and vincristine [40]. Using ideal body weight for dosing in obesity may result in individual underdosing, hence in cases where no differences in the distribution of antineoplastics between obese and non-obese individuals exist, dose-adjustment based on actual body weight appears to reduce the risk of underdosing [61].

### Effect of nutritional status on pharmacokinetics

#### Undernutrition

No conclusion on the overall impact of undernutrition on antineoplastic pharmacokinetics can be made from the available studies. Some reports indicate pharmacokinetic alterations (increased AUC and decreased clearance) for vincristine [40], methotrexate [36, 45, 48, 50] and doxorubicin [34]. However, cutoffs used to define undernutrition differed significantly per study population and no studies investigated the effect of severe malnutrition (which often accompanies a childhood cancer diagnosis in LMIC). Within the context of (severe) undernutrition, no studies defined or delineated the possible impact of protein-energy malnutrition or energy malnutrition, on pharmacokinetics, particularly in children from LMIC where there is a greater likelihood of concurrent severe chronic, or severe-acute malnutrition, compared to HIC [62–64]. Additional considerations in undernutrition, such as the impact of micronutrient deficiencies, particularly the fat-soluble vitamins (namely vitamins A, D, & E) on pharmacokinetic variability were not explored [63, 65].

Studies from India and Malawi show increased drug exposure, altered clearance, increased AUC of vincristine [40, 66], and decreased clearance and increased elimination half-life of methotrexate [45]; however, their effects on clinical outcomes were not reported. Moreover, small sample sizes in these studies limit comparisons, and protein-energy malnutrition has known effects on kidney filtration and liver function (decreased) which also impacts pharmacokinetics [67]. Undernutrition has been shown to lead to increased methotrexate toxicity during maintenance therapy due to reduced bone marrow reserves [23] and prolonged neutropenia [68]. In comparison, in a larger sample of children in the United States of America (*n* = 621) no effect of decreased BMI on methotrexate clearance was reported; however this cohort of children did not experience any severe undernutrition [48]. More recently, Vincristine has been studied in greater detail in Kenyan children (without malnutrition) [66], where despite higher exposure to vincristine compared to European patients, these children did not develop vincristine-induced peripheral neuropathy [66]. It is unlikely that pharmacokinetic differences account for this, highlighting the need to consider genetic variation alongside nutritional status and possible pharmacodynamics which were not analysed in this population [66].

Undernutrition, especially protein-energy malnutrition, reduces hepatic metabolism, increases urinary excretion, and elevates AUC for doxorubicin in rats [69]. Both undernutrition and anthracycline administration are cardiotoxic in animal models, suggesting that dose adjustments may be necessary [70]. Only two pharmacokinetic studies for doxorubicin reported increased peak plasma concentration and decreased clearance among undernourished children, therefore no association with outcomes can be made [34, 43]. Neither of these studies included severely malnourished children [34, 43]. There is a direct need to evaluate pharmacokinetics among children with severe undernutrition in context of cancer, due to the high burden in LMIC, their inferior outcomes, and a lack of data to support dosing strategies in the context of cancer.

#### Overweight and obesity

The impact of overweight and obesity on antineoplastic pharmacokinetics is likely with doxorubicin [34, 42, 46], methotrexate [36], mercaptopurine [44], and busulfan [39]. However, conclusions on their overall pharmacological impact are difficult to make due to variations in the classification of obesity (BMI-for-age ≥95th percentile, percentage body fat and/or body composition). For example, doxorubicin clearance and distribution were similar across patients classified by BMI-for-age percentile or percent body fat [41–43, 46]. Yet Thompson et al. described altered clearance and AUC in children with >30% body fat [46], an important consideration for cardiotoxicity risk. Sex-based pharmacokinetic differences are noted in anthracyclines among adult and adolescent populations, where females have a lower plasma drug clearance, resulting in an increased risk of cardiotoxicity and lower survival outcomes. However, variations of body fat by sex remain largely unexplored [71, 72]. Discrepancies in obesity classification underscore the need for standardized approaches to body composition assessment.

Mercaptopurine concentrations were subtherapeutic in obese children using dosing based on actual body weight [44]. However, despite documenting a strong negative correlation (r = −0.75) between BMI-for-age percentile and AUC, this prospective study had a small sample size (*n* = 18) [44]. In addition, the definition used for higher fat mass (BMI-for-age percentile of >75th), was not in agreement with the accepted cut-off for obesity (BMI-for-age ≥95th percentile) according to the World Health Organisation [24]. Using the internationally recognised cut-off values BMI-for-age ≥95th percentile, Hijiya et al. did not find any correlation between obesity and drug clearance [48]. Obese children also exhibited supratherapeutic busulfan levels [39]. Dose calculation recommendations from the two studies on busulfan agreed on the use of dose according to actual body weight [39, 49]. Both subtherapeutic and supratherapeutic drug exposure have clinical ramifications, increasing the potential for under-treating a malignancy (lowering treatment response) or the risk of toxicity, respectively.

### Nutritional status and outcomes

The correlation between nutritional status and outcomes in paediatric cancer patients is well-documented [71–76]. Obesity has been linked to increased treatment-related mortality in AML and decreased overall survival and 5-year event-free survival in ALL and severely undernourished children have overall inferior outcomes and increased toxicity risk [66, 73, 74]. However, data on nutritional status, pharmacokinetics and outcomes in paediatric cancer are limited and vary by drug, and few studies have investigated drug disposition in these patients. Three PK studies explored associations between nutritional status and outcomes [34, 35, 44]. There were no differences in survival or toxicity for methotrexate, cytarabine, etoposide, or teniposide based on BMI-for-age percentiles [48]. Associations between Doxorubicin and cardiotoxicity have been reported in those with a lower mean BMI (continuous variable) and cumulative doses >200 mg/m2 [34], or those with protein-calorie malnutrition (with a particular focus on females with systolic dysfunction) [77]. For methotrexate, a delayed elimination in obese (body fat) and large (i.e. body size) patients, necessitating extended rescue therapies as intravenous leucovorin, alkalinised hydration and prolonged hospital admissions were described without any reported toxicity risk [36]. Among obese children, higher rates of neurotoxicity associated with vincristine were reported previously [78–80]. Enhanced reporting on standardized pharmacokinetic parameters combined with outcomes is crucial for further understanding these possible associations and improving outcomes for children with cancer globally.

### Pharmacogenetics

Single nucleotide polymorphisms are increasingly recognized for their role in antineoplastic pharmacokinetic variability. To date, anthracyclines have the most data available among children in terms of pharmacogenetic relevance [34, 37]. Thompson et al. found associations between reduced daunorubicin clearance and FMO3 (a hepatic microsomal enzyme) and GSTP1 (a glutathione S-transferase) haplotypes, independent of nutritional status [37]. Pharmacogenetic data are predominantly available for Caucasian populations, yet studies suggest its importance in evaluating pharmacokinetics in diverse ethnicities. African American children exhibited reduced vincristine toxicity due to increased CYP3A5 expression [81] and in a sample of Brazilian patients’ renal function and body size had a greater influence on Methotrexate clearance compared to genetics [50]. Despite lacking pharmacogenetic analysis, integrating nutritional status and genotype in future studies is imperative for understanding systemic circulation, distribution volume, therapeutic range, and toxicity risk associated with the narrow therapeutic windows of chemotherapeutics.

### Strengths & limitations

This review was performed according to the PRISMA framework [26], ensuring a comprehensive search strategy that yielded more studies meeting inclusion criteria compared to prior reviews [61, 82, 83]. The inclusion of terms including ‘undernutrition’ and ‘overweight’ expanded the study scope by five [34, 38, 39, 44, 50] and seven [34–38, 42, 84] studies, respectively. Limitations include few studies per antineoplastic dosing regimen (eight drugs only had one study available), heterogeneous definitions of nutritional status, small sample sizes, neglect of ethnicity and genetics (potentially confounding observed pharmacokinetic differences across populations) and low-quality evidence (Supplementary Table 3). In addition, while there was information at each end of the spectrum of under- and overnutrition, only six studies further defined the extremes of either nutritional state, which may have underestimated the effect on pharmacokinetics. Lastly, no information was available on pharmacodynamics in all included studies.

### Recommendation & conclusion

This review highlights the lack of evidence and consensus on the impact of nutritional status and body composition on antineoplastic pharmacokinetics and pharmacodynamics in paediatric cancer patients, and subsequent dosing implications. Nutritional status definitions, pharmacokinetic analyses and dosing strategies, varied widely among included studies. The SIOP Nutrition Network recommends standardisation of nutritional assessment methods, using international cutoffs for undernutrition and obesity. Also, height/length and weight are used to calculate BMI and MUAC should be used to determine body composition (where more sophisticated measures are not available), and when there is a significant tumour load, making weight-based measurements less reliable. Sensitive body composition measures beyond BMI (such as percent body fat) and diverse ethnic representation are crucial for global correlation. Larger, standardized prospective studies incorporating ethnicity, pharmacogenetics, and consistent pharmacokinetic parameter generation are warranted to bridge this knowledge gap and facilitate global data pooling. Outside of clinical studies, regular monitoring of chemotherapy blood/plasma concentrations is recommended, particularly in children who are severely underweight or obesity. Finally, the standardization of pharmacokinetic assessment within treatment protocols could enhance future data aggregation.

## Supplementary information

Supplement material

Prisma Checklist
