# Supplementary material for: Nutritional status, body composition and chemotherapy dosing in children and young people with cancer: a systematic review by the SIOP nutrition network
Source: Br J Cancer. 2025 Jun 26;133(3):275–85. doi: 10.1038/s41416-025-03023-3 (PMC12322071; doi:10.1038/s41416-025-03023-3)
Supplement: Supplementary file 1 — Supplement material [file 41416_2025_3023_MOESM1_ESM.docx]

| **Description of Tables of Figures** | **Page** |
| --- | --- |
| **Supplementary 1**. Proposed search terms prepared for MEDLINE database | 2 |
| **Supplementary 2**. Definition of nutritional status used for each of the included studies and classification of participants. | 3 |
| **Supplementary 3.** Pharmacokinetic methodology and key outcomes with Risk of Bias and GRADE assessment for each antineoplastic drug included in the review. | 5 |

**Supplementary 1**. Proposed search terms prepared for MEDLINE database.

| **Population** | Child/ [MeSH], Pediatrics/ [MeSH], Adolescent/ [MeSH], Infant/ [MeSH] (child* or pediatric or pediatric or adolescen* or teen* or kids or preteen or pre-teen or youth or infan* or "young adult*" or "school age*").mp. |
| --- | --- |
|  | Neoplasms [MeSH] (neoplasm* or cancer or “p?ediatric oncology” or “p?ediatric cancer” or “child* cancer” ).mp. |
| **Intervention** | Drug Therapy/ [MeSH], Antineoplastic Agents/ [MeSH], Alkylating Agents/ [MeSH], Antineoplastic Combined Chemotherapy Protocols/ [MeSH] (chemotherap* or "drug therapy" or "antineoplastic agent*" or "alkylating agent*" or cytotox* or antitumo?r or anti?cancer).mp. |
| **Comparison** | Malnutrition/ [MeSH], Thinness/ [MeSH], Protein-Energy Malnutrition/ [MeSH], Severe Acute Malnutrition/ [MeSH], sarcopenia/ [MeSH] (malnutrition or thinness or "protein-energy malnutrition" or "severe-acute malnutrition" or malnourish* or underweight or PEM or "protein-calorie malnutrition" or "nutritional deficien*" or undernutrition or sarcopeni*).mp. |
|  | Overnutrition/ [MeSH], Overweight/ [MeSH], Obesity/ [MeSH] (overnutrition or overweight or obesity or obese or hypernutrition or “sarcopenic obesity”).mp. |
|  | Nutritional Status/ [MeSH], Body Composition/ [MeSH], Body Mass Index/ [MeSH], Body Weight/ [MeSH]  ("nutrition* status" or "body composition" or "body mass index" or "body weight" or BMI).mp. |
| **Outcome** | Pharmacokinetics/ [MeSH], Toxicology/ [MeSH], Metabolism/ [MeSH], Pharmacogenetics/ [MeSH] (pharmacokinetic* or toxic* or metabolism or pharmacodynamic).mp. |
|  | “Drug-Related Side Effects and Adverse Reactions”/ [MeSH], (“drug toxicity” or “drug side effect*” or toxicit*).mp, |
|  | Survival/ [MeSH], Disease-Free Survival/ [MeSH], Survival Rate/ [MeSH], Progression-Free Survival/ [MeSH], Survival Analysis/ [MeSH]  (surviv* or “disease-free surviv*” or “survival rate” or “progression-free surviv*” or “survival analysis”).mp |

**Keyword search**: [mp=title, book title, abstract, original title, name of substance word, subject heading word, floating sub-heading word, keyword heading word, organism supplementary concept word, protocol supplementary concept word, rare disease supplementary concept word, unique identifier, synonyms, population supplementary concept word, anatomy supplementary concept word]

**Supplementary 2**. Definition of nutritional status used for each of the included studies and classification of participants.

| **Author**  **Year of publication** | **Nutrition status** | | | | |
| --- | --- | --- | --- | --- | --- |
|  | **Definition Used** | **Undernutrition** | **Normal nutrition** | **Overweight** | **Obesity** |
| Egnell et al. 2023 [47] | Cole et al. for underweight [55]  IOTF Guidelines for overweight/obesity [56] | <17 kg/m^2^ | 18.5 - <25 kg/m^2^ | 25 – 30 kg/m^e^ | ≥30 kgm/^2^ |
| de Oliveira Henz et al. [50] | WHO z-score for age [54] | Split into two  -2 (very underweight)  -1 - -2 (underweight | -1 – 1 (normal weight) | 1 – 2 (overweight) | >2 (very overweight) |
| Gandara-Mireles et al. 2022 [34] | BMI (kg/m^2^) | BMI ≤19.4 or ≥19.4 | | | |
| Gibson et al.  2021 [35] | CDC BMI-for-age [50, 51] | <5^th^ centile (n=0) | 5–84.9^th^ centile (n=17) | 85–94.9^th^ centile (n=9) | 85–94.9^th^ centile (n=10) |
| Orgel et al.  2021 [36] | CDC BMI-for-age [50, 51] | < 95^th^ centile (n=30) | ≥ 95^th^ centile (n=6) | | |
|  | Body fat percentage (DXA) | < 45% (n=29) | ≥ 45% (n=7) | | |
| Thompson et al.  2014 [37] | CDC BMI-for-age [50, 51] | <10^th^ centile (n=5) | 10-85^th^ centile (n=65) | 85-95^th^ centile (n=12) | >95^th^ centile (n=16) |
|  | Body fat percentage (DXA) | ≤30% (n=33); >30% (n=15) | | |  |
| Turner et al.  2014 [38] | CDC BMI-for-age [50, 51] | <5^th^ centile (n=3) |  | ≥85^th^ centile (n=11) |  |
| Browning et al.  2011 [39] | CDC BMI-for-age [50, 51] | <25^th^ centile (n=17) | 25-85^th^ centile (n=29) | ≥85^th^ centile (n=22) |  |
|  | CDC BMI-for-age (extremes) | 0-5^th^ centile (n=6) | >5^th^-95^th^ centile (n=49) | | ≥95^th^ centile (n=13) |
| Israels et al. 2010 [40] | 78 NCHS growth curve (HANES data) [80] | Expressed as z-score for corrected weight-for-height | | | |
| Thompson et al.  2009 [46] | CDC Z-scores [50, 51]  CDC BMI-for-age [50, 51] | <10^th^ centile (n=5) | 10^th^ – 85^th^ centile (n=15) | ≥85^th^ centile (n=2) | |
|  | Body fat percentage (DXA) | ≤30% (n=16); >30% (n=6) | | | |
| Ritzmo et al.  2007 [51] | BMI (kg/m^2^) |  |  |  | 46.3 kg/m^2^ (n=2) |
| Hijiya et al.  2006 [48] | CDC BMI-for-age [50, 51] | ≤10^th^ centile (n=102) | 10^th^ – 85^th^ centile (n=400) | ≥85^th^ - <95^th^ centile (n=64) | ≥95^th^ centile (n=55) |
| Hempel et al.  2002 [41] | BMI (kg/m^2^) | Presented as a continuous variable | | | |
| Frost et al  2002 [42] | BMI (kg/m^2^) | Presented as a continuous variable | | | |
| Dupuis et al.  2000 [49] | Actual body weight  Ideal body weight [81]  Effective body weight [81] | Presented as a continuous variable | | | |
| Eksborg et al.  2000 [43] | BMI (kg/m^2^)  Lean Body Mass (LBM) [82] | Presented as a continuous variable | | | |
| Zuccaro et al.  1991 [44] | Weight/height centile | <75^th^ centile | | >75^th^ centile | |
| Kumar et al.  1987 [45] | Relative weight | Presented as a continuous variable | | | |

**Supplementary 3.** Pharmacokinetic methodology and key outcomes with Risk of Bias and GRADE assessment for each antineoplastic drug included in the review.

| **Author** | **Method** | | **Number of patients n (%)** | | | | | | | **Findings** | | **Risk of bias [ref]** | |  |
| --- | --- | --- | --- | --- | --- | --- | --- | --- | --- | --- | --- | --- | --- | --- |
|  |  |  | **Undernutrition** | | **Normal nutrition** | | **Overweight** | | **Obesity** |  |  |  |  |  |
| **Bevacizumab** | | | | | | | | | | | |  | |  |
| Turner et al, 2014 [38] | Population PK model  BMI% a significant covariate for model. | | 3 (1) | | 13 (48) | | 11 (41) | | - | Body composition a key determinant of exposure.  BMI% was significantly (P<0.05) correlated to body weight normalized clearance and volume of distribution.  V_normalised_ of bevacizumab was up to 53% lower (BMI > 97th percentile) or up to 32.1% higher (BMI < 3rd percentile) than the population median value.  BW to be a suboptimal body size descriptor for PK in children. | | SB: high  AB: low  DB: high  CF: low | |  |
| ***GRADE assessment***  Study design:  Study limitations:  Consistency:  Directness:  Precision:  Publication bias:  Effect size  Dose response:  Plausible confounding: | +2  -1  0  0  -2  0  0  0  0 | Observational evidence.  Limitations: Selection bias high in 0/1; Attrition bias low in 1/1; Detection bias high in 1/1; Confounding low in 1/1.  One study available.  Results are direct. Population and outcomes broadly generalizable.  Important imprecision. Small sample size. Only one study identified.  Publication bias unlikely.  No large magnitude of effect.  Evidence of body composition being a key determinant of exposure. Only one study identified.  Confounders adjusted for in the PK model. | | | | | | | | | | | |  |
| ***Quality of evidence:*** ⊕⊖⊖⊖ Very low-quality evidence  ***Conclusion:*** Body composition is related to **v**ariability in bevacizumab exposure (clearance and volume of distribution) using weight-based dosing in children with osteosarcoma.  (1 study demonstrating this effect, 27 participants, 80 PK samples, one population PK analysis) | | | | | | | | | | | | | |  |
| **Busulfan** | | | | | | | | | | | |  | |  |
| Browning et al, 2011 [39] | Dosing study.  PK of test dose (TD) used to determine daily dose of Busulfan.  Regimen dose (RD) altered based on PK of TD. | | 11 (30) | | NR^a^ | | 18 (49) | | NR | High BMIs had higher AUCs when dosing on actual weight compared to BMI (higher BMI required less drug).  Higher BMI had lower Busulfan clearance and drug requirements (drug dose/kg) to achieve the same AUC as normal or low BMI.  No association between BMI category and being under/over target for TD AUC.  Using adjusted-BW, 53% of patients with high BMIs would have an AUC under/over target.  Patients in extreme low BMI category (<5^th^ centile) required the highest dose. | | SB: high  AB: low  DB: high  CF: low | |  |
| Dupuis et al, 2000 [49] | Dose adjustment PK study. | | BMI not reported. Calculated actual, ideal, and effective weight. | | | | | | | Obesity did not significantly affect busulfan dose requirements. | | SB: high  AB: low  DB: high  CF: unclear | |  |
| ***GRADE assessment***  Study design:  Study limitations:  Consistency:  Directness:  Precision:  Publication bias:  Effect size  Dose response:  Plausible confounding: | +2  -2  -1  0  -1  0  0  0  0 | Observational – one prospective and one retrospective.  Limitations: Selection bias high in 2/2; Attrition bias low in 2/2; Detection bias high in 2/2; Confounding low in 1/2.  Lack of agreement between studies.  Population and outcomes broadly generalizable.  Small sample sizes (22 participants with BMI ≥85^th^ percentile in one study), not all participants had a malignancy in one study, small effect size)  Publication bias unlikely.  No large effect size.  Evidence of higher exposure in higher BMI in one study. Heterogeneity for dose requirements.  Confounders adjusted for in the PK model (observational study). | | | | | | | | | | | |  |
| ***Quality of evidence:*** ⊕⊖⊖⊖ Very low-quality evidence  ***Conclusion***: Heterogeneity for dose requirements and obesity (2 studies, inconsistent conclusions, 75 participants, 397 PK samples, two dose adjustment analyses). | | | | | | | | | | | | | |  |
| **Crizotinib** | | | | | | | | | | | |  | |  |
| Gibson, 2021 [35] | Steady-state PK model  Two-compartment pop PK model  AUC and C_max_ adjusted for nutritional status, concomitant dasatinib | | 0 (0) | | 17 (47) | | 9 (25) | | 10 (28) | Overweight/obese patients exhibited lower CL/F (mean 44.2 L/h/m^2^) compared to normal weight (mean 75.5 L/h/m^2^; p=0.0015).  Overweight and obese patients had 1.5-fold increase in AUC_SS,0-24_.  Overweight and obese patients had 1.6-fold increase C_max,SS_. | | SB: high  AB: low  DB: high  CF: low | |  |
| ***GRADE assessment***  Study design:  Study limitations:  Consistency:  Directness:  Precision:  Publication bias:  Effect size  Dose response:  Plausible confounding: | +2  -2  0  0  -2  0  0  0  0 | Observational evidence.  Limitations: Selection bias high in 1/1; Attrition bias low in 1/1; Detection bias high in 1/1; Confounding low in 1/1.  One study available.  Population and outcomes broadly generalizable.  Important imprecision. Small sample size. Only one study identified.  Publication bias unlikely.  No large effect size.  Evidence of higher exposure in overweight/obese participants. Only one study identified.  Confounders adjusted for in the PK model (observational study). | | | | | | | | | | | |  |
| ***Quality of evidence:*** ⊕⊖⊖⊖ Very low-quality evidence  ***Conclusion:*** Higher crizotinib exposure in overweight/obese patients (1 study, 36 participants, 74 PK samples, one single dose and steady state PK analysis). | | | | | | | | | | | | | |  |
| **Cytarabine** |  | |  | |  | |  | |  |  | |  | |  |
| Hijiya et al., 2006 [48] | One-compartment PK model  Model adjusted for age, course, study. | | 102 (16) | | 400 (64) | | 64 (11)^b^ | | 55 (9)^c^ | Mean systemic clearance of the underweight, risk of overweight, and overweight groups differed from that of the normal-weight group by less than 17% (P>0.3)  No effect of nutritional status on clearance. | | SB: low  AB: low  DB: high  CF: low | |  |
| ***GRADE assessment***  Study design:  Study limitations:  Consistency:  Directness:  Precision:  Publication bias:  Effect size  Dose response:  Plausible confounding: | +2  -1  0  0  -1  0  0  0  0 | Observational evidence.  Limitations: Selection bias low in 1/1; Attrition bias low in 1/1; Detection bias high in 1/1; Confounding low in 1/1.  One study available.  Population and outcomes broadly generalizable.  Important imprecision. Large sample size. Only one study identified.  Publication bias unlikely.  No large effect size.  No evidence of effect of nutritional status on outcomes.  Confounders adjusted for in the PK model (observational study). | | | | | | | | | | | |  |
| ***Quality of evidence:*** ⊕⊖⊖⊖ Very low quality evidence  ***Conclusion:*** No relationship between nutritional status and cytarabine clearance (1 study, 621 participants, 5,020 courses, 1 multivariable PK analysis) | | | | | | | | | | | | | |  |
| **Dasatinib** | | | | | | | | | | | |  | |  |
| Gibson, 2021 [35] | Steady-state PK model  Two-compartment pop PK model  AUC and C_max_ adjusted for nutritional status, concomitant dasatinib | | 0 (0) | | 17 (47) | | 9 (25) | | 10 (28) | Concomitant dasatinib administration in overweight/obese participants resulted in a clinically relevant impact (>20%) on drug exposure.  Concomitant dasatinib resulted in 0.64-fold decrease in AUC_SS0-24_  Concomitant dasatinib resulted in 0.69-fold decrease in C_max,ss_ | | SB: high  AB: low  DB: high  CF: low | |  |
| ***GRADE assessment***  Study design:  Study limitations:  Consistency:  Directness:  Precision:  Publication bias:  Effect size  Dose response:  Plausible confounding: | +2  -2  0  0  -2  0  0  0  0 | Observational evidence.  Limitations: Selection bias high in 1/1; Attrition bias low in 1/1; Detection bias high in 1/1; Confounding low in 1/1.  One study available.  Population and outcomes broadly generalizable.  Important imprecision. Small sample size. Only one study identified.  Publication bias unlikely.  No large effect size.  Evidence of effect of concomitant dasatinib administration and higher exposure in overweight/obese participants. Only one study identified.  Confounders adjusted for in the PK model (observational study). | | | | | | | | | | | |  |
| ***Quality of evidence:*** ⊕⊖⊖⊖ Very low quality evidence  ***Conclusion:*** Administering dasatinib with crizotinib resulted in a reduction in exposure in overweight/obese patients compared to crizotinib alone (1 study, 36 participants, 74 PK samples, one single dose and steady state PK analysis). | | | | | | | | | | | | | |  |
| **Daunorubicin (and daunorubicinol)** | | | | | | | | | | | |  | |  |
| Thompson et al, 2014 [37] | Two-compartment PK model - Daunorubicin.  One-compartment PK model – Daunorubicinol.  PK of Daunorubicin fixed to estimate parameters. | | 5 (5) | | 65 (66) | | 12 (12) | | 16 (16) | No effect of body composition (BF%) or BMI% on PK parameters observed. | | SB: low  AB: low  DB: high  CF: low | |  |
| ***GRADE assessment***  Study design:  Study limitations:  Consistency:  Directness:  Precision:  Publication bias:  Effect size  Dose response:  Plausible confounding: | +2  -1  0  0  -2  0  0  0  0 | Observational evidence.  Limitations: Selection bias low in 1/1; Attrition bias low in 1/1; Detection bias high in 1/1; Confounding low in 1/1.  One study available.  Population and outcomes broadly generalizable.  Important imprecision. Small sample size. Only one study identified.  Publication bias unlikely.  No large effect size.  No evidence of effect of nutritional status on outcomes.  Confounders adjusted for in the PK model (observational study). | | | | | | | | | | | |  |
| ***Quality of evidence:*** ⊕⊖⊖⊖ Very low quality evidence  ***Conclusion:*** No effect of body composition on PK of daunorubicin and its metabolite, daunorubicinol (1 study, 98 participants, total number of samples not stated, 1 multivariate PK analysis) | | | | | | | | | | | | | |  |
| **Doxorubicin (and Doxorubicinol)** | | | | | | | | | | | |  | |  |
| Gándara-Mireles, 2022 [34] | Two-compartment model  PK parameters linearly scaled as a function of BSA. | | BMI expressed as a continuous variable. | | | | | | | Low BMI (<19.4 kg/m^2^) associated with decreased clearance (r=-0.873; p=0.003).  Low BMI (<19.4 kg/m^2^) associated with a decrease in intercompartmental clearance [Q], (r=-0.997; p=0.002).  Low BMI (<19.4 kg/m^2^) associated with decreased peripheral volume compartment [V2], (r=−0.872;p=0.04). | | SB: high  AB: unclear  DB: high  CF: low | |  |
| Thompson, 2009 [46] | Three-compartment model (Doxorubicin)  One-compartment model (Doxorubicinol)  PK linearly scaled based on BSA | | 5 (23) | | 15 (68) | | 2 (9) | | - | **Doxorubicin**  BF% ≥30% had lower clearance than patients with BF% <30% (64.8 L/m^2^ vs. 37.2 L/m^2^, respectively; p=0.033).  **Doxorubicinol**  BF% ≥30% volume of distribution lower than in patients with BF% <30% (802 vs. 1,450 L/m^2^; p=0.021).  Volume of distribution lower in overweight patients compared to normal weight or underweight (NS) according to BMI percentile. | | SB: low  AB: low  DB: high  CF: low | |  |
| Ritzmo, 2007 [51] | Limited sampling PK model (one patient).  Data compared with literature. | | - | | - | | - | | 46.3 kg/m^2 d^ | Plasma clearance comparable with non-obese pediatric patients. | | SB: high  AB: low  DB: high  CF: unclear | |  |
| Frost, 2002 [42] | Limited sampling model  Observed concentrations normalised for a dose of 40 mg/m^2^  PK adjusted for WBC and BMI | | BMI expressed as a continuous variable. | | | | | | | **Doxorubicin**  No significant correlation between doxorubicin concentration and BMI.  No effect of BMI on PK data for children >1 year of age.  **Doxorubicinol**  No effect of BMI on PK data for children >1 year of age. | | SB: high  AB: low  DB: high  CF: low | |  |
| Hempel, 2002 [41] | Three-compartment PK model.  Sampled peak plasma concentration. | | BMI expressed as a continuous variable: 16.7 (13.7-22.2) k/m^2^ | | | | | | | No correlation between C_max_ and BMI. | | SB: high  AB: low  DB: high  CF: low | |  |
| Eksborg, 2000 [43] | Limited sampling PK model.  Dose normalisation performed. | | NR | NR | | NR | | NR | | C_max_ didn’t correlate with LBM  Dose-normalised C_max_ higher in patients with low BMI (12.2-16.3 kg/m^2^), 1.33 vs. 1.21 mg/m^2^ (p=0.06) compared to high BMI (16.7-21.5 kg/m^2^). | | SB: high  AB: low  DB: high  CF: unclear | |  |
| ***GRADE assessment***  Study design:  Study limitations:  Consistency:  Directness:  Precision:  Publication bias:  Effect size  Dose response:  Plausible confounding: | +2  -2  -1  0  -1  0  0  0  0 | Observational evidence (6 studies)  Limitations: Selection bias high in 5/6; Attrition bias low in 5/6; Detection bias high in 6/6; Confounding low in 4/6.  Lack of consistent results.  Indirectness does not appear to be an issue.  Some imprecisions. Small cumulative sample size.  Publication bias unlikely.  No large effect size.  No clear dose-response relationship.  Confounders adjusted for in the PK model | | | | | | | | | | | |  |
| ***Quality of evidence:*** ⊕⊖⊖⊖ Very low-quality evidence  ***Conclusion:*** Inconsistent relationship between BMI or body composition (LBM) and PK. (6 studies, 258 participants, total number of samples not stated, 6 multivariate analyses) | | | | | | | | | | | | | |  |
| **Epidoxorubicin** (Epirubicin) | | | | | | | | | | | |  | |  |
| Eksborg, 2000 [43] | Limited sampling PK model.  Dose normalisation performed. | | NR | | NR | | NR | | NR | C_max_ didn’t correlate with LBM.  Dose-normalised C_max_ higher in patients with low BMI (12.2-16.3 kg/m^2^), 0.98 vs. 0.89 mg/m^2^ (p=0.02) compared to high BMI (16.7-21.5 kg/m^2^) | | SB: high  AB: low  DB: high  CF: unclear | |  |
| ***GRADE assessment***  Study design:  Study limitations:  Consistency:  Directness:  Precision:  Publication bias:  Effect size  Dose response:  Plausible confounding: | +2  -2  0  0  -2  0  0  0  0 | Observational evidence.  Limitations: Selection bias high in 1/1; Attrition bias low in 1/1; Detection bias high in 1/1  One study available.  Population and outcomes broadly generalizable.  Important imprecision. Small sample size. Only one study identified.  Publication bias unlikely.  No large effect size.  Only one study identified.  Confounders adjusted for in the PK model (observational study). | | | | | | | | | | | |  |
| ***Quality of evidence:*** ⊕⊖⊖⊖ Very low-quality evidence  ***Conclusion:*** Dose-normalised max concentration higher in low BMI (1 study, 31 participants, total number of samples not stated, non-parametric PK analysis) | | | | | | | | | | | | | |  |
| **Etoposide** | | | | | | | | | | | |  | |  |
| Ritzmo, 2007 [51] | Limited sampling PK model (one patient).  Data compared with literature. | | - | | - | | - | | 46.3 kg/m^2 d^ | Plasma clearance comparable with non-obese pediatric patients. | |  | |  |
| Hijiya, 2006 [48] | Two-compartment PK model  Model adjusted for age, course, study. | | 102 (16) | | 400 (64) | | 64 (11)^b^ | | 55 (9)^c^ | Mean systemic clearance of the underweight, risk of overweight, and overweight groups differed from that of the normal-weight group by less than 17% (P>0.3)  No effect of nutritional status on clearance. | | SB: low  AB: low  DB: high  CF: low | |  |
| ***GRADE assessment***  Study design:  Study limitations:  Consistency:  Directness:  Precision:  Publication bias:  Effect size  Dose response:  Plausible confounding: | +2  -2  0  0  -2  0  0  0  0 | Observational evidence.  Limitations: Detection bias high in 1/1. The other study is a case study.  One study available + case study  Population and outcomes broadly generalizable for one study.  Important imprecision. Small sample size. Only one study identified.  Publication bias unlikely.  No large effect size.  Only one study identified.  Confounders adjusted for in the PK model (observational study). | | | | | | | | | | | |  |
| ***Quality of evidence:*** ⊕⊖⊖⊖ Very low-quality evidence  ***Conclusion:*** one study available as other study is a case study (n=1). No impact of nutritional status on clearance (1 study, 621 participants, retrospective, number of samples not reported,1 multivariate PK analysis) | | | | | | | | | | | | | |  |
| **Mercaptopurine** | | | | | | | | | | | | |  | |
| Zuccaro, 1991 [44] | Standard equations. | | BMI% divided into <75^th^ percentile (n=9) and >75^th^ percentile (n=9). | | | | | | | | Higher AUC in children <75^th^ percentile (867.3 vs. 357.1 ng/mL/h; p<0.001).  Significant correlation between AUC and BM% percentiles (r=-0.75)  Children with BMI percentile >95^th^ (obese) had lower serum concentrations.  Higher clearance in children >75^th^ percentile (20.9 vs. 93.4 l/h; p<0.001).  Higher volume of distribution in children >75^th^ percentile (236.3 vs. 142.4 L/kg; p=0.05).  Lower C_max_ in children >75^th^ percentile (140.8 vs. 323.4 ng/mL; p=<0.001) | | SB: high  AB: low  DB: high  CF: unclear | |
| Hijiya, 2006 [48] | Two-compartment PK model  Model adjusted for age, course, study. | | 102 (16) | | 400 (64) | | 64 (11)^b^ | | 55 (9)^c^ | | No effect of nutritional status on clearance | | SB: low  AB: low  DB: high  CF: low | |
| ***GRADE assessment***  Study design:  Study limitations:  Consistency:  Directness:  Precision:  Publication bias:  Effect size  Dose response:  Plausible confounding: | +2  -2  -1  -1  -1  0  0  0  0 | Observational evidence.  Limitations: Selection bias high in 1/2; Detection bias high in 2/2  Lack of consistency between studies  Population and outcomes broadly generalizable for one study.  Important imprecision. Small sample size in one study  Publication bias unlikely.  No large effect size.  Only in one study  Confounders adjusted for in the PK model (observational study) in one study | | | | | | | | | | | | |
| ***Quality of evidence:*** ⊕⊖⊖⊖ Very low-quality evidence  ***Conclusion:*** Inconsistent relationship between nutritional status and PK. (2 studies, 639 participants, number of samples not reported, 1 multivariate PK analysis) | | | | | | | | | | | | | | |
| **Methotrexate** | | | | | | | | | | | | |  | |
| Egnell et al. 2023 [47] | NR | | 9 (5) | | 146 (80) | | 20 (11) | | 7 (4) | | Higher concentrations and prolonged excretion in obese children at diagnosis (35.4% of courses in obese patients had levels ≥1 µM/L compared to 18.5% in normal weight and 1-% in overweight; p=0.003.  Independently significant PK effect for children with a decrease in BMI SDS before course 1 of treatment. | | SB: low  AB: low  DB: high  CF: low | |
| de Oliveira Henz et al. 2023 [50] | Two-compartment model | | 4 (10) | | 26 (57) | | 7 (16) | | 4 (10) | | Low classification of BMI z-score of -2 and between -1 and -2 showed reduced clearance compared to those with a higher BMI. | | SB: low  AB: low  DB: high  CF: low | |
| Orgel et al., 2021 [36] | Non-parametric population PK model  Dosing scaled to BSA. | | NR | | NR | | NR | | 6 (17) by BMI%  7 (19) by BF% | | Body fat percent (obesity) associated with 2-fold increased risk for delayed elimination at 48 hrs.  Holding body fat constant, children with higher BSA had a similarly increased risk for delayed elimination.  After accounting for BSA (body size), BF% influenced the PK of HDMTX.  BSA and BF% were not linearly associated with increased AUC (p=0.74 and p=0.12).  Population PK modelling: BSA and BF% were independently correlated with higher HDMTX at 48 hours. | | SB: high  AB: high  DB: high  CF: low | |
| Hijiya et al., 2006 [48] | Two-compartment PK model  Model adjusted for age, course, study. | | 102 (16) | | 400 (64) | | 64 (11)^b^ | | 55 (9)^c^ | | No effect of nutritional status on clearance. | | SB: low  AB: low  DB: high  CF: low | |
| Kumar et al., 1987 [45] | Standard formula used to estimate PK parameters.  Based on relative weight being a good indication of nutrition status in adults. | | 5 (83) | | 1 (17) | | - | | - | | Relative weight was negatively correlated with elimination half-life.  Volume of central compartment and tissue compartment were not associated with relative weight.  Plasma albumin showed good correlation with relative weight, and albumin negatively correlated with elimination half-life. | | SB: low  AB: low  DB: high  CF: unclear | |
| ***GRADE assessment***  Study design:  Study limitations:  Consistency:  Directness:  Precision:  Publication bias:  Effect size  Dose response:  Plausible confounding: | +2  -2  -1  -1  -1  0  0  0  0 | Observational evidence.  Limitations: Selection bias high in 1/6; Attrition bias in 1/6. Detection bias high in 6/6.  Lack of consistency between studies  Population and outcomes broadly generalizable  Important imprecision. Small sample size in two studies.  Publication bias unlikely.  No large effect size.  Only in one study. No response in one study. not clear in the other.  Confounders adjusted for in the PK model (observational study) in one study | | | | | | | | | | | | |
| ***Quality of evidence:*** ⊕⊖⊖⊖ Very low quality evidence  ***Conclusion:*** Inconsistent relationship between nutritional status and PK. (5 studies, 890 participants, total number of samples not reported, 4 multivariate PK analyses) | | | | | | | | | | | | | | |
| **Teniposide** | | | | | | | | | | | | |  | |
| Hijiya, 2006 [48] | Two-compartment PK model  PK model adjusted for age, course, study. | | 102 (16) | | 400 (64) | | 64 (11)^b^ | | 55 (9)^c^ | | No effect of nutritional status on clearance. | | SB: low  AB: low  DB: high  CF: low | |
| ***GRADE assessment***  Study design:  Study limitations:  Consistency:  Directness:  Precision:  Publication bias:  Effect size  Dose response:  Plausible confounding: | +2  -1  0  0  -1  0  0  0  0 | Observational evidence.  Limitations: Selection bias low in 1/1; Attrition bias low in 1/1; Detection bias high in 1/1; Confounding low in 1/1.  One study available.  Population and outcomes broadly generalizable.  Important imprecision. Large sample size. Only one study identified.  Publication bias unlikely.  No large effect size.  No evidence of effect of nutritional status on outcomes.  Confounders adjusted for in the PK model (observational study). | | | | | | | | | | | | |
| ***Quality of evidence:*** ⊕⊖⊖⊖ Very low quality evidence  ***Conclusion:*** 1 study demonstrating no effect of nutritional stats on PK (1 study, 621 participants, total number of samples not reported, 1 multivariate PK analysis) | | | | | | | | | | | | | | |
| **Vincristine** | | | | | | | | | | | | |  | |
| Israels, 2010 [40] | Non-compartmental analysis. | | 11 (58) | | 8 (42) | | - | | - | | Mean clearance lower in undernourished (Malawian) patients (121.3 vs. 361 mL/min).  Mean log clearance lower in undernourished (Malawian) patients (2.2 mL/min/m^2^ vs. 2.6 mL/min/m^2^; p=0.001).  1.98-fold larger mean log Vincristine AUC in undernourished (Malawian) patients (3.8 µg/mL/min vs. 3.5 µg/mL/min; p=0.003).  A decrease in -1 z-score associated with +0.061 (p=0.043) change in log_10_ AUC.  The difference in AUC values was statistically significantly explained by nutritional status (p= 0.043). | | SB: high  AB: low  DB: high  CF: low | |
| ***GRADE assessment***  Study design:  Study limitations:  Consistency:  Directness:  Precision:  Publication bias:  Effect size  Dose response:  Plausible confounding: | +2  -1  0  -1  -1  0  0  +1  0 | Observational evidence.  Limitations: Selection bias high in 1/1; Attrition bias low in 1/1; Detection bias high in 1/1; Confounding low in 1/1.  One study available.  small sample size. Different ethnicities compared.  Important imprecision. small sample size. Only one study identified.  Publication bias unlikely.  No large effect size.  Clear evidence of effect of nutritional status on outcomes.  Confounders adjusted for in the PK model (observational study). | | | | | | | | | | | | |
| ***Quality of evidence:*** ⊕⊖⊖⊖ Very low quality evidence  ***Conclusion:*** 1 study available demonstrating lower clearance in undernourished patients (1 study,19 participants, total number of samples not reported, regression analysis) | | | | | | | | | | | | | | |

^a^ Number of normal weight participants with a malignancy not reported; ^b^ Defined as ‘at risk of overnutrition’; ^c^ Defined as overweight; ^d^ based on one patient

**Abbreviations**: AUC, area under the curve; BSA, body surface area; BMI, body mass index, BMI%, body mass index percentile; HDMTX, high-dose methotrexate; NR, not reported; PK, pharmacokinetics; TD, treatment dose; WBC, white blood cells
